# Supplementary material for: Channelized topography amplifies melt-sensitivity of cold Antarctic ice shelves
Source: Nat Commun. 2026 May 7;17:3790. doi: 10.1038/s41467-026-71828-8 (PMC13153326; doi:10.1038/s41467-026-71828-8)
Supplement: Supplementary file 1 — Supplementary Information [file 41467_2026_71828_MOESM1_ESM.pdf]

# Supplementary Information for

# Channelized topography amplifies melt-sensitivity of cold Antarctic ice shelves

Qin Zhou[1*†], Tore Hattermann[2*†], Chen Zhao[3], Rupert Gladstone[4], Julius Lauber[2,5], Petteri Uotila[6], and Ashley Morris[7]

[1]Akvaplan-niva AS, Tromsø, Norway
[2]Norwegian Polar Institute, Tromsø, Norway
[3]Australian Centre for Excellence in Antarctic Science & Australian Antarctic Program Partnership, Institute for Marine and Antarctic Studies, University of Tasmania, Hobart, Australia
[4]Arctic Centre, University of Lapland, Rovaniemi, Finland
[5]Multiconsult, Tromsø, Norway
[6]Institute for Atmospheric and Earth System Research/Physics, University of Helsinki, Helsinki, Finland
[7]Svalbard Integrated Arctic Earth Observing System, Longyearbyen, Norway
[†]These authors contributed equally.
*qin.zhou@akvaplan.niva.no
*tore.hattermann@npolar.no

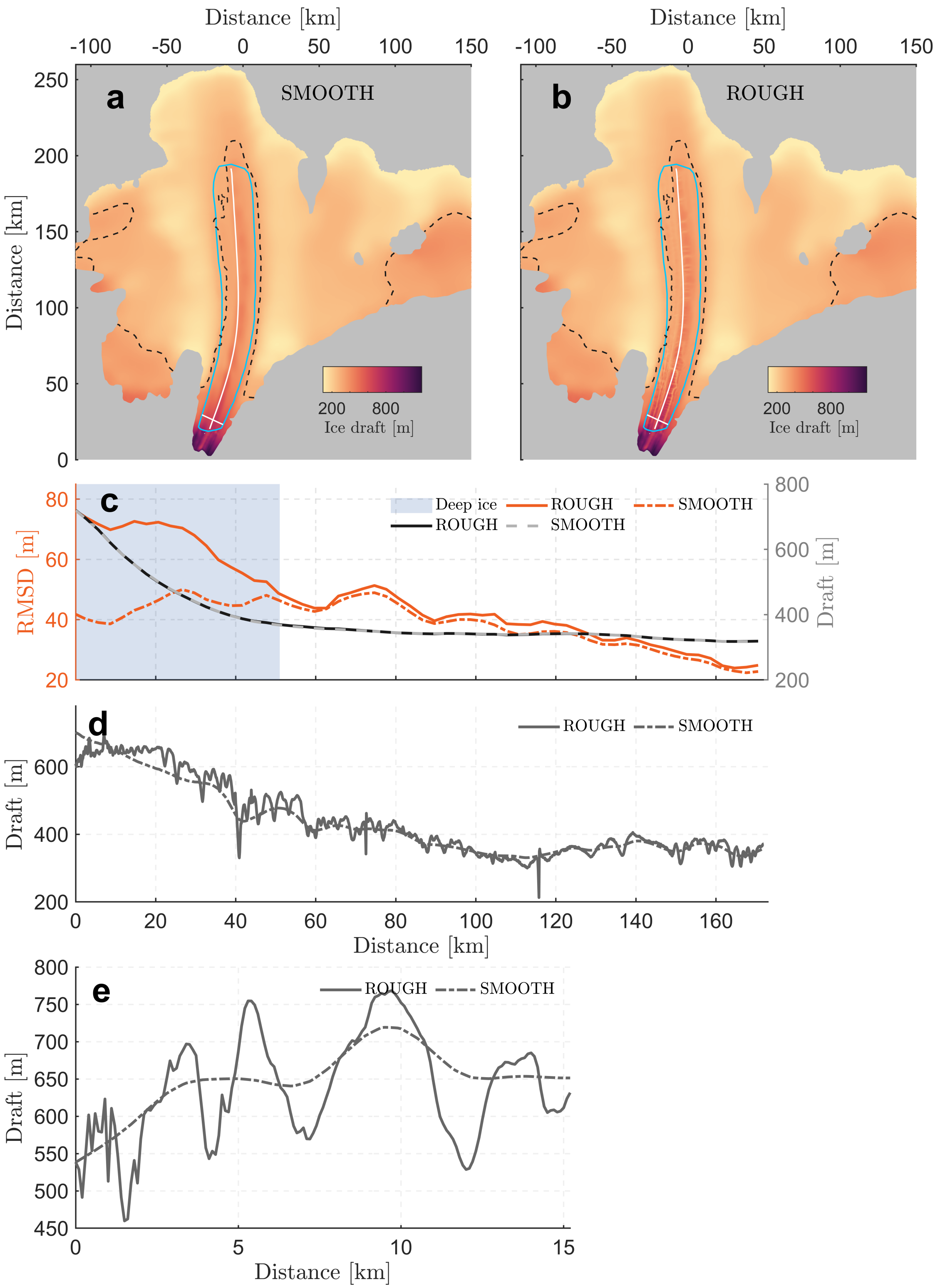


**Figure S1.** **Difference between the SMOOTH and ROUGH ice drafts. a, b,** SMOOTH (a) and ROUGH (b) ice drafts used in our experiments. Black dashed lines indicate the 300 m ice draft contour, and the blue polygon outlines the smoothing region. White lines indicate transects used for comparisons in panels **d, e**. **c,** Cross-stream-averaged Root Mean Square Deviation (RMSD) of the SMOOTH and ROUGH ice drafts (left axis) and ice drafts (right axis) along the ice stream. **d,** ROUGH (solid line) and SMOOTH (dashed line) ice draft profiles along the along-stream transect. **e,** ROUGH (solid line) and SMOOTH (dashed line) ice draft profiles along the cross-stream transect. Distance in **c, d** is measured from the southernmost point of the smoothing region, and in **e** from the westernmost point of the transect.

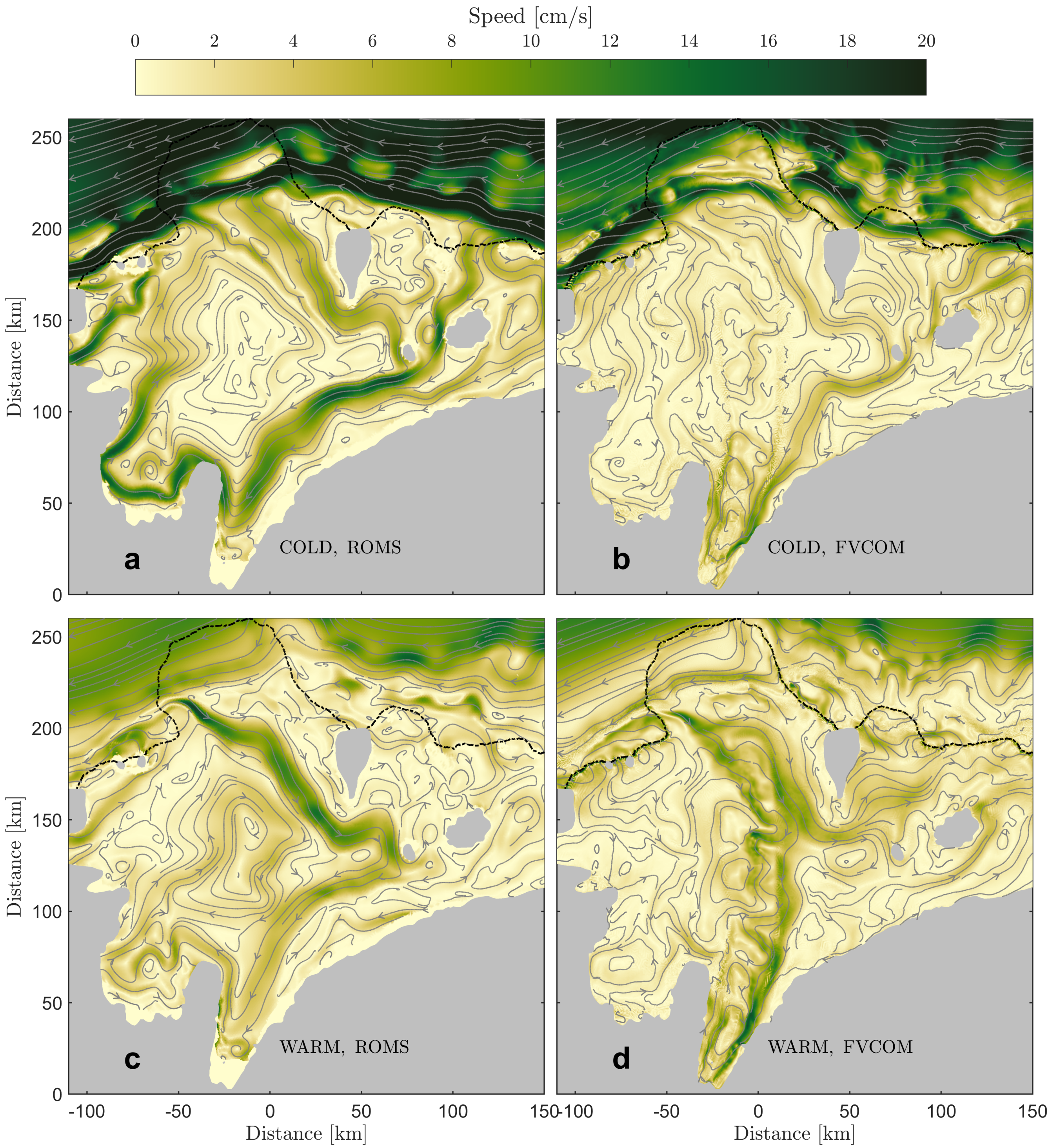


**Figure S2. Bottom circulation comparison. a, b,** Bottom velocity fields from the ROMS [1] (a) and FVCOM (b) Fimbulisen ice shelf-ocean models under COLD ocean forcing. **c, d,** Bottom velocity fields from ROMS (c) and FVCOM (d) under WARM forcing. ROUGH ice drafts are used in the FVCOM results. ROMS results shown here are interpolated onto the FVCOM grid. Black dashed lines mark the ice front.

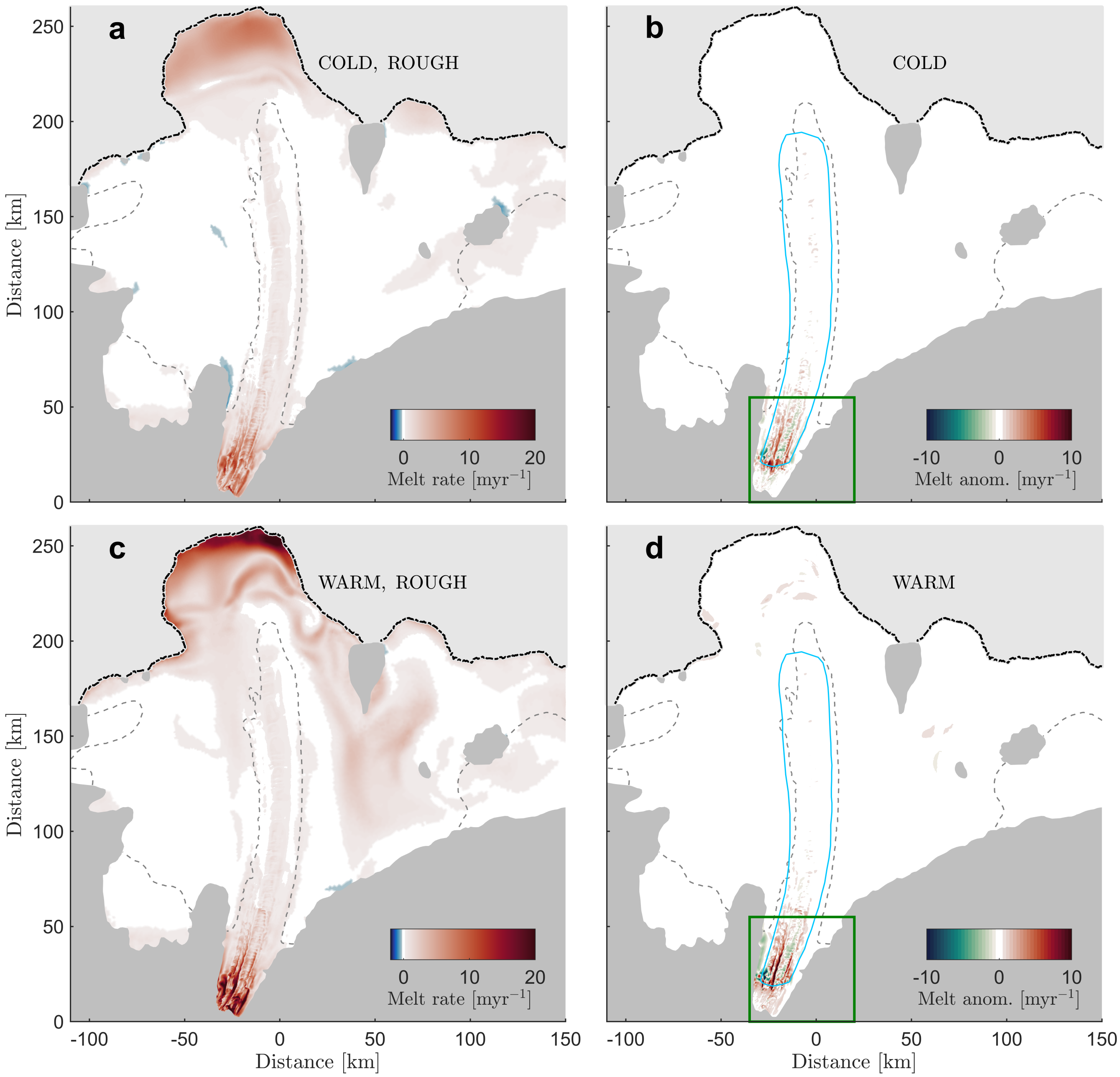


**Figure S3.** **Melt rates and melt rate anomalies. a, c,** Spatial distribution of melt rates in the ROUGH draft experiments under COLD (a) and WARM (c) forcing. **b, d,** Spatial distribution of melt rate anomalies in the ROUGH draft experiment relative to the SMOOTH draft under COLD (b) and WARM (d) forcing. Black dashed lines mark the ice front, and grey dashed lines mark the 300 m ice draft contour. Blue polygons in **b,d** indicate the smoothing region, and green rectangles indicate the deep-ice region.

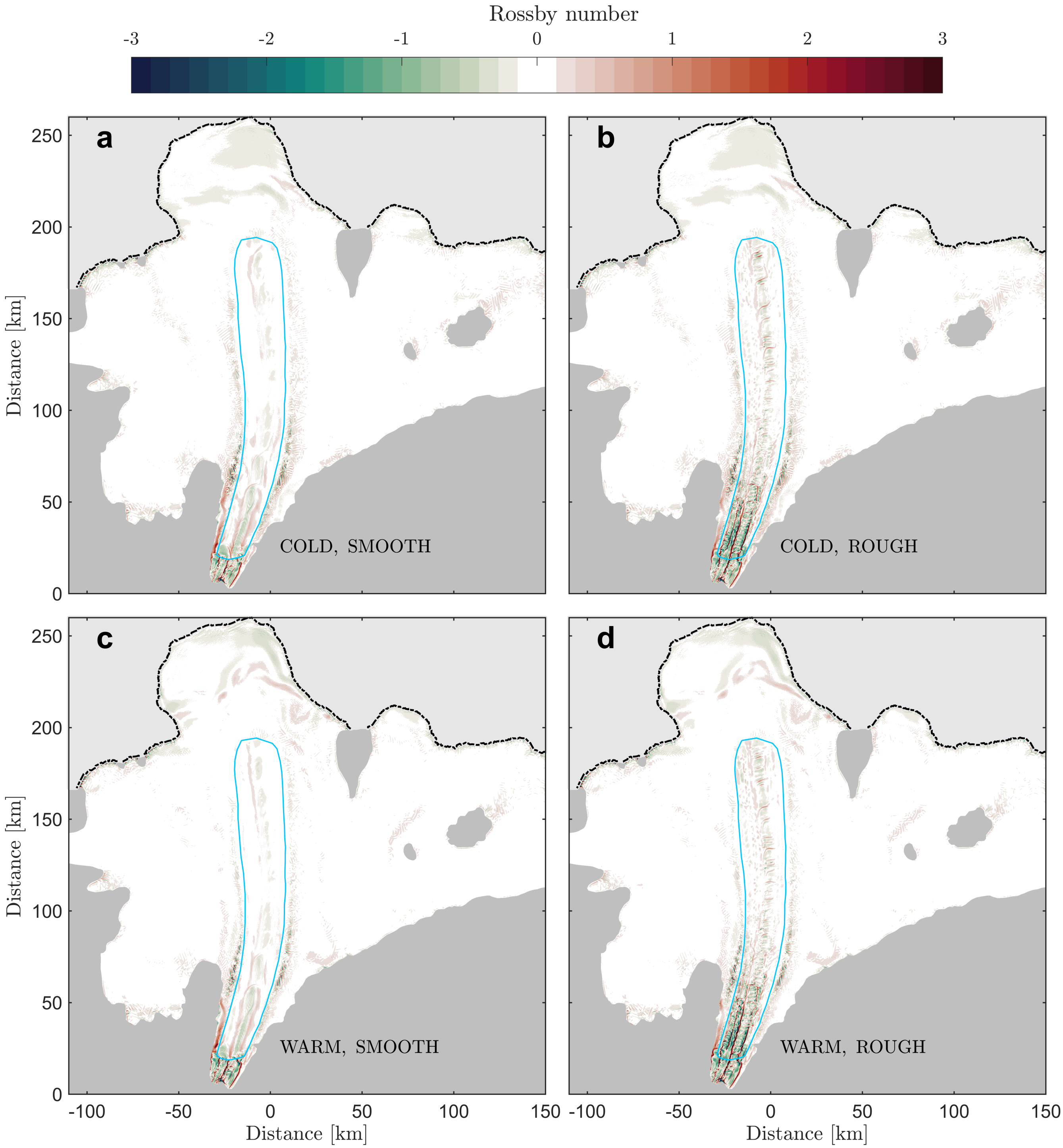


**Figure S4.** **Submesoscale eddy variability. a, b,** Spatial distribution of Rossby numbers near the ice base in the SMOOTH (a) and ROUGH (b) draft experiments under COLD forcing. **c, d,** Spatial distribution of Rossby numbers near the ice base in the SMOOTH (c) and ROUGH (d) draft experiments under WARM forcing. Black dashed lines mark the ice front. Blue polygons indicate the smoothing region.

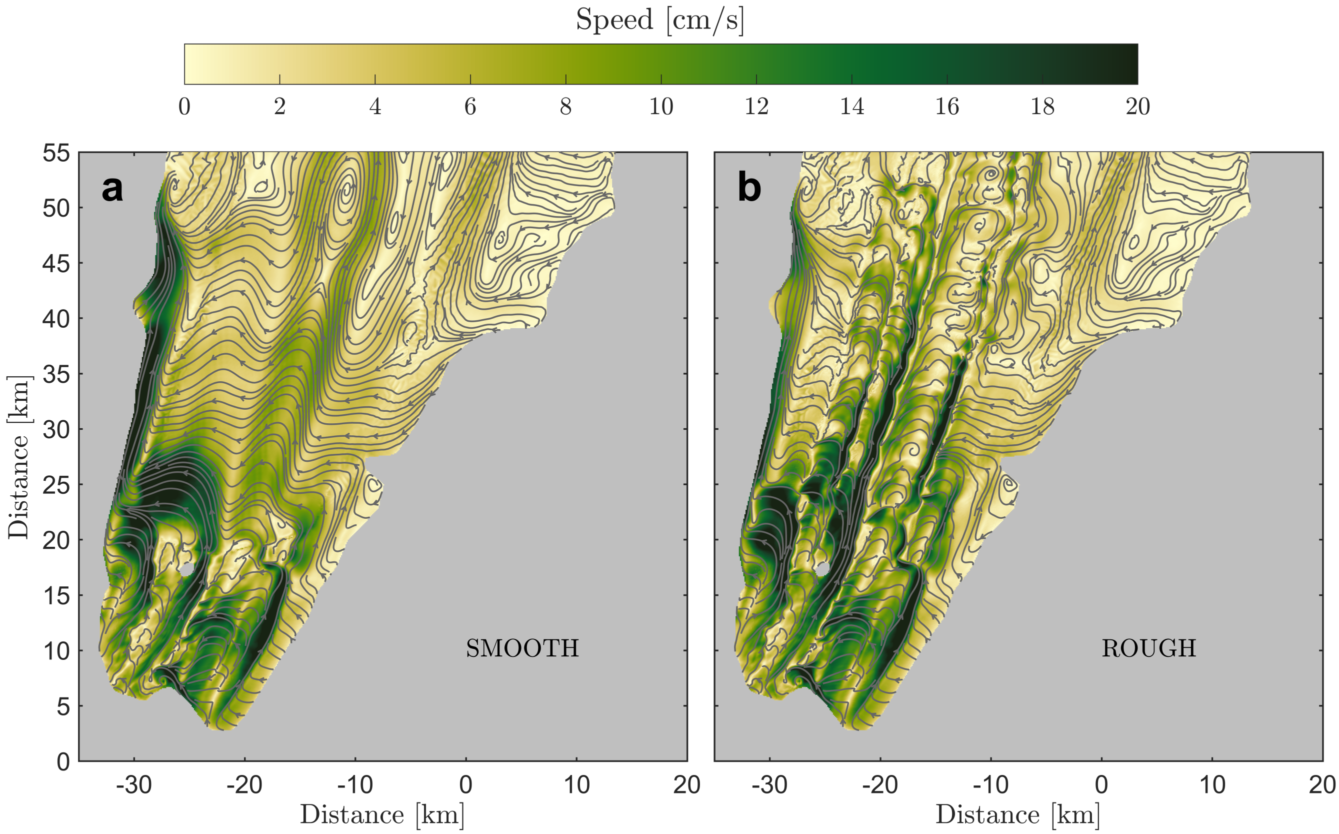


**Figure S5.** **Circulations near the ice base in the deep-ice region. a, b,** Surface velocity fields from the SMOOTH (a) and ROUGH (b) draft experiments under WARM forcing.

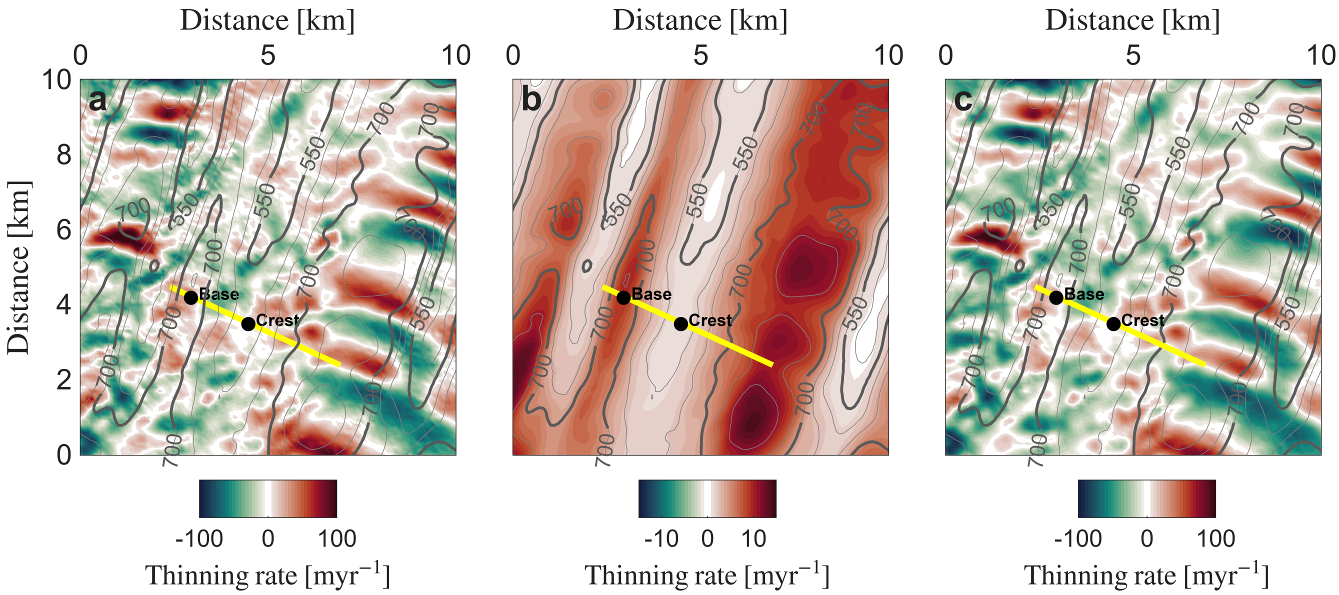


**Figure S6.** **Ice shelf thinning rates produced by Fimbulisen ice dynamic model. a, b, c,** Spatial distribution of the total thinning rates (a), the mean deformational thinning rates (b), and the advection-induced thinning rates (c) within a $10 \times 10$ km box surrounding the example channel (thick yellow line). Black dots indicate the channel base and crest. Gray contours indicate the ice draft at 50 m intervals. Advection-induced thinning rates are shown for completeness but are excluded from the comparison with ocean-driven melt rates, which uses deformation-induced thinning rates only.

## References

1. Hattermann, T., Smedsrud, L., Nøst, O., Lilly, J. & Galton-Fenzi, B. Eddy-resolving simulations of the Fimbul Ice Shelf cavity circulation: Basal melting and exchange with open ocean. *Ocean. Model.* **82**, 28–44, DOI: 10.1016/j.ocemod.2014.07.004 (2014).
